# 

**Published:** 1864-09

**Authors:** 


*Contents.*

ORIGINAL COMMUNICATIONS ........ 129

EDITORIAL ........ 138

CHRONICLE OF MEDICAL SCIENCE ........ 140

OBITUARY ........ 147

INTERESTING ANALYSES OF ARTICLES IN FOREIGN JOURNALS ........ 147

